# 2321. Long-term immunogenicity of Ad26.RSV.preF/RSV preF protein vaccine against RSV in a phase 2b study by age and risk level

**DOI:** 10.1093/ofid/ofac492.152

**Published:** 2022-12-15

**Authors:** Christy A Comeaux, Ann R Falsey, Kristi Williams, Stephan Bart, John E Ervin, Arangassery R Bastian, Joris Menten, Els De Paepe, Sjouke Vandenberghe, Eric K H Chan, Jerald Sadoff, Macaya Douoguih, Benoit Callendret, Esther Heijnen

**Affiliations:** Janssen Vaccines & Prevention B.V., Leiden, Zuid-Holland, Netherlands; University of Rochester School of Medicine, Rochester, New York; Janssen Research and Development, Spring House, Pennsylvania; Trial Professional Consultant Group, Woodstock, Maryland; AMR Kansas City, Kansas City, Missouri; Janssen Vaccines & Prevention BV, Leiden, Zuid-Holland, Netherlands; Janssen Infectious Diseases, Beerse, Antwerpen, Belgium; Janssen Infectious Diseases, Beerse, Antwerpen, Belgium; Janssen Infectious Diseases, Beerse, Antwerpen, Belgium; Janssen Global Services, LLC, Raritan, New Jersey; Janssen Vaccines & Prevention BV, Leiden, Zuid-Holland, Netherlands; Janssen Vaccines & Prevention B.V., Leiden, Zuid-Holland, Netherlands; Janssen Vaccines & Prevention B.V., Leiden, Zuid-Holland, Netherlands; Janssen Vaccines & Prevention B.V., Leiden, Zuid-Holland, Netherlands

## Abstract

**Background:**

Respiratory syncytial virus (RSV) can cause serious lower respiratory tract disease (LRTD) among older adults. There is no licensed RSV vaccine. In CYPRESS (a randomized, double-blind, placebo-controlled, phase 2b proof-of concept trial; NCT03982199), an Ad26.RSV.preF/RSV preF protein vaccine demonstrated 80.0% efficacy for prevention of RSV LRTD and 69.8% efficacy for prevention of any RSV acute respiratory infection in adults aged ≥65 years through the first RSV season. This study evaluated the durability of immune responses elicited by Ad26.RSV.preF/RSV preF protein after two RSV seasons (up to 1.5 years post-vaccination) in the overall study population and in groups of participants stratified by age and risk level for severe RSV LRTD.

**Methods:**

Participants (N=5782) were randomized 1:1 to receive vaccine or placebo before the RSV season. The primary endpoint was first occurrence of RSV LRTD. RSV A2 virus neutralizing antibodies (VNAs; through Day 365), RSV preF binding antibodies (through Day 533), and RSV-F–specific IFN-γ enzyme-linked immune absorbent spot (ELISpot; through Day 533), were evaluated in an immunogenicity subset (n=195; ages 65–74 years: n=141; 75–84 years: n=47; ≥85 years: n=6; increased risk [chronic heart or lung disease]: n=48; non-increased risk: n=147).

**Results:**

In the vaccine group of the immunogenicity subset, RSV A2 VNAs peaked at Day 15 and were maintained at 2.8-fold over baseline at 1 year. Similarly, RSV preF-specific binding antibodies peaked at Day 15 and were maintained at 2.1-fold above baseline at 1.5 years. Median RSV-F–specific IFN-γ T-cell frequency increased from 34 spot-forming cells (SFC)/10^6^ peripheral blood mononuclear cells (PBMCs) at baseline to 143 SFC/10^6^ PBMCs at 1.5 years. Comparable immune responses were observed in age/risk subgroups. No relevant changes were observed in the placebo group at any time point. Pre-existing Ad26 VNAs did not appear to impact RSV-specific immune response durability.

**Conclusion:**

Ad26.RSV.preF/RSV preF protein vaccine was efficacious and elicited robust, durable (to at least 1.5 years) humoral and cellular immune responses in adults aged ≥65 years, older participants (≥75 years), and in participants with increased risk for severe RSV LRTD.

**Disclosures:**

**Christy A. Comeaux, MD, PhD**, Janssen Vaccines & Prevention B.V.: Employee **Ann R. Falsey, MD**, BioFire Diagnostics: Grant/Research Support|Janssen: Grant/Research Support|Merck Sharp & Dohme: Grant/Research Support|Novavax: Advisor/Consultant|Pfizer: Grant/Research Support **Kristi Williams, PhD**, Janssen Research and Development: Employee **John E. Ervin, MD**, The Alliance for Multispecialty Research – KCM: Contractual agreement for conduct of study protocol **Arangassery R. Bastian, PhD**, Janssen Vaccines & Prevention BV: Employee **Joris Menten, PhD**, Janssen Infectious Diseases: Employee **Els De Paepe, MSc**, Janssen Infectious Diseases: employee **Sjouke Vandenberghe, PhD**, Janssen Infectious Diseases: Employee **Eric K. H. Chan, PhD**, Janssen Global Services, LLC: Employee **Jerald Sadoff, MD**, Janssen Vaccines & Prevention BV: Employee **Macaya Douoguih, MD, MPH**, Janssen Vaccines & Prevention B.V.: Employee **Benoit Callendret, PhD**, Janssen Vaccines & Prevention B.V.: Employee **Esther Heijnen, MD, PhD**, Janssen Vaccines & Prevention B.V.: Employee.

